# Management of Neovascular Age-Related Macular Degeneration in Clinical Practice: Initiation, Maintenance, and Discontinuation of Therapy

**DOI:** 10.1155/2011/752543

**Published:** 2011-11-22

**Authors:** Pearse A. Keane, Adnan Tufail, Praveen J. Patel

**Affiliations:** NIHR Biomedical Research Centre for Ophthalmology, Moorfields Eye Hospital NHS Foundation Trust, City Road, London EC1V 2PD, UK

## Abstract

Neovascular age-related macular degeneration (AMD) is a leading cause of irreversible visual loss in elderly populations. In recent years, pharmacological inhibition of vascular endothelial growth factor (VEGF), via intravitreal injection of ranibizumab (Lucentis) or bevacizumab (Avastin), has offered the first opportunity to improve visual outcomes in patients diagnosed with this disorder. In this paper, we provide recommendations on how bevacizumab and ranibizumab may be best applied in current clinical practice, with an emphasis on their underlying pharmacology and efficacy. In addition, we review current guidelines for the initiation, maintenance, and discontinuation of anti-VEGF therapies, as well as emerging treatment strategies and future directions in the field.

## 1. Introduction

Despite the recent introduction of new therapeutic approaches, and an improved awareness of modifiable disease risk factors, age-related macular degeneration (AMD) remains a leading cause of irreversible visual loss in elderly populations [1]. In the neovascular form of this disorder, severe visual loss commonly occurs as a result of the invasion of abnormal blood vessels from the choroidal circulation with damage to the overlying retina (choroidal neovascularization (CNV)) [2]. In recent years, pharmacological inhibition of this process, through blockade of the proangiogenic glycoprotein, vascular endothelial growth factor (VEGF), has offered the first opportunity to improve visual outcomes in patients diagnosed with this disorder [3].

In 2006, the results of the MARINA and ANCHOR studies demonstrated that inhibition of VEGF by frequent treatment with ranibizumab (Lucentis, Genentech, Inc., South San Francisco, CA) could lead to significant visual improvement in patients with neovascular AMD [4, 5]. However, while these pivotal clinical trials were still underway, Rosenfeld et al. provided evidence that an agent designed for treatment of colorectal cancer—bevacizumab (Avastin, Genentech)—could also lead to significant visual gains, but at a substantially lower cost [6]. Consequently, bevacizumab was quickly adopted for the treatment of neovascular AMD by clinicians worldwide [7–9]. 

The widespread adoption of bevacizumab for the treatment of neovascular AMD occurred in the absence of formal guidelines from clinical trials; as a result, clinicians had limited evidence for their initial efforts at followup and retreatment [10, 11]. “As required” retreatment strategies, based on the assessment of disease activity using optical coherence tomography (OCT), thus evolved, and their success quickly led to their adoption for use with ranibizumab [12]. While variable retreatment regimens offer a number of potential advantages, they place a greater onus on the clinician for accurate, and consistent, clinical assessment over extended time periods—an obligation made difficult with the rapid evolution of retinal imaging techniques and the proliferation of treatment strategies.

In this paper, we address these concerns by providing recommendations on how bevacizumab and ranibizumab may be best applied in current clinical practice. We begin by providing an overview of bevacizumab and ranibizumab, with an emphasis on pharmacology and efficacy. We next review current guidelines for the initiation, maintenance, and discontinuation of anti-VEGF therapies. We conclude with a review of emerging treatment strategies and future directions in the field.

## 2. Ranibizumab

### 2.1. Pharmacology

Ranibizumab (formerly known as rhuFAb V2) is an antibody fragment that binds and inhibits all isoforms of VEGF [13]. It is a chimeric molecule, consisting of an antigen-binding murine component, and a nonbinding human component that serves to make it less antigenic. Ranibizumab was developed by alteration of bevacizumab, a humanized version of a murine monoclonal antibody first derived in 1996 (the decision to alter bevacizumab in this manner was driven by preclinical studies suggesting that a full-size antibody would be unable to penetrate the retina) [13]. Substitution of targeted amino acids was also performed in a bid to maximize the binding affinity of ranibizumab for VEGF, in the hope that this change would lead to improved outcomes (the VEGF binding affinity of ranibizumab is approximately 100 times that of bevacizumab) [13].

Intravitreously administered ranibizumab is thought to exit the vitreous cavity via one of two pathways: posteriorly via retinal penetration and then drainage into the choroidal vasculature or anteriorly through the aqueous drainage route [14]. Knowledge of the vitreous half-life is an important consideration when optimizing retreatment frequencies, whereas serum concentrations are an important factor with respect to systemic adverse effects (e.g., stroke). The pharmacokinetics of ranibizumab, after intravitreous administration, have thus been studied both in animal models and in human trials [15–17]. In animal studies, ranibizumab is cleared from the vitreous with a half-life of approximately three days. After reaching a maximum at approximately one day, the serum concentration of ranibizumab declines in parallel with this. In human studies, following monthly intravitreous ranibizumab administration, maximum serum concentrations were dose dependent but low (0.3 ng/mL to 2.36 ng/mL—levels thought to be below the concentrations necessary for reduction in biological activity of VEGF by 50%). Based on neovascular AMD population pharmacokinetic analysis, maximum serum concentrations of 1.5 ng/mL are predicted to be reached at approximately one day after monthly intravitreous administration of 0.5 mg of ranibizumab (in humans, serum ranibizumab concentrations are predicted to be approximately 90,000-fold lower than vitreous concentrations). Thus, based on its elimination from serum, the estimated average vitreous elimination half-life was approximately nine days (http://www.gene.com/gene/products/information/pdf/lucentis-prescribing.pdf).

### 2.2. Efficacy

In 2006, ranibizumab was licensed for use in the United States following publication of the MARINA and ANCHOR studies [4, 5]. 

In the MARINA trial, patients with either “minimally classic” or “occult” angiographic leakage patterns were randomized to receive monthly injections of intravitreous ranibizumab (0.3 mg or 0.5 mg) or monthly sham injections [5]. At the 12-month point of this study, visual acuity had improved by 15 or more letters in 24.8% of the 0.3 mg group and 33.8% of the 0.5 mg group (as compared with only 5.0% of the sham-injection group). Furthermore, patients receiving ranibizumab, on average, demonstrated increases in visual acuity (6.5 letters in the 0.3 mg group and 7.2 letters in the 0.5 mg group), while those receiving sham therapy, on average, demonstrated losses (10.4 letters in the sham-injection group). In addition, the vast majority of patients receiving ranibizumab avoided moderate visual loss (94.5% of the group receiving 0.3 mg of ranibizumab and 94.6% of those receiving 0.5 mg), while only 62.2% of the control group managed to do so. Subsequently, at the 24-month conclusion of the study, the visual benefits gained from the ranibizumab groups were maintained.

In the ANCHOR trial, patients with “predominantly classic” angiographic leakage patterns were randomized to receive either 24 monthly intravitreous injections of ranibizumab (either 0.3 mg or 0.5 mg) or photodynamic therapy with verteporfin [4]. Visual acuity improved by 15 letters or more in 35.7% of the 0.3 mg group and 40.3% of the 0.5 mg group (as compared with 5.6% of the verteporfin group). As in the MARINA trial, those receiving ranibizumab also demonstrated a mean increase in visual acuity (8.5 letters in the 0.3 mg group and 11.3 letters in the 0.5 mg group), while those in the control group experienced a mean decrease (9.5 letters in the verteporfin group). Similarly, 94.3% of those given 0.3 mg of ranibizumab and 96.4% of those given 0.5 mg avoided further moderate visual loss (as compared with 64.3% of those in the verteporfin group). 

Subgroup analyses were also performed in both the MARINA and ANCHOR studies and demonstrated the importance of prompt diagnosis and treatment in patients with neovascular AMD—that is, younger patients, with smaller lesions, and relatively preserved visual acuities, tended to have the best visual outcomes [18, 19]. Conversely, patients with moderate visual loss after monthly treatment with ranibizumab were found to have a higher concentration of atrophic scarring and pigmentary abnormalities, suggesting the need for additional strategies aimed at photoreceptor and retinal pigment epithelium (RPE) cell preservation [20].

## 3. Bevacizumab

### 3.1. Pharmacology

Bevacizumab is a full-length monoclonal antibody, first derived from a murine source and prepared for intravenous administration, which binds to and inhibits the biologic activity of all isoforms of VEGF [13, 14]. Bevacizumab was originally developed and approved for the treatment of metastatic colorectal cancer, but it is now also used in the treatment of nonsmall cell lung cancer and in patients with metastatic breast cancer [21]. The results of preliminary studies suggested that bevacizumab, due to its large size, would not be fully distributed within the retinal layers—hence, the development of an antibody fragment, ranibizumab, for the treatment of neovascular AMD [13, 14]. Furthermore, the longer half-life of a complete antibody, versus an antibody fragment, raised concerns regarding systemic toxicity in patients requiring long-term anti-VEGF blockade.

In comparison with ranibizumab, less is known about the intraocular pharmacokinetics of bevacizumab. In a recent study by Bakri et al., a rabbit model was used to study the vitreous half-life and serum concentrations after intravitreous injection of 1.25 mg of bevacizumab [22]. In this study, the vitreous half-life was found to be longer than ranibizumab at 4.32 days (versus 2.88 days for ranibizumab), and the maximum serum concentrations were reached after eight days. Of note, small amounts of bevacizumab were detected in the vitreous of the fellow, uninjected eye. In an animal study (albino rabbits) by Shahar et al., confocal immunohistochemistry was used to demonstrate full thickness retinal penetration 24 hours after intravitreous injection (and the essential absence of bevacizumab) by four weeks after injection [23]. In a more recent study performed in humans, an aqueous half-life of 9.82 days was found after intravitreous injection of 1.5 mg of bevacizumab [24].

### 3.2. Efficacy

In 2005, Rosenfeld et al. reported that intravitreous administration of bevacizumab led to rapid resolution of abnormalities on OCT in a patient with neovascular AMD [6]. A large number of off-label, short-term, uncontrolled, retrospective case series have since evaluated the efficacy of intravitreous bevacizumab in neovascular AMD—until recently, however, no evidence from randomized controlled clinical trials existed for its use [25]. 

In 2010, the results of the ABC trial provided the first level I evidence for the efficacy of intravitreous bevacizumab in neovascular AMD [26, 27]. In this prospective, double-masked, multicenter, randomized, controlled trial, 32% of patients treated with bevacizumab gained 15 or more letters from baseline visual acuity. More than 45% of patients treated with bevacizumab improved 10 or more letters, a threshold that exceeds the variability of the measurement of visual acuity and represents the proportion of patients recovering vision. In addition, 91% of patients receiving bevacizumab lost fewer than 15 letters of visual acuity from baseline (i.e., avoided moderate visual loss). Finally, mean visual acuity increased by 7.0 letters in the bevacizumab group with a median of seven injections over a 54-week period.

In 2011, the results of the CATT study provided further level I evidence for the efficacy of bevacizumab in the treatment of neovascular AMD [28]. In this study, patients with neovascular AMD were randomized to receive intravitreous injections of ranibizumab or bevacizumab on either a fixed, monthly schedule, or on an “as required” basis. The results of this study suggest that bevacizumab administered monthly is equivalent to ranibizumab administered monthly (mean visual acuity increase of 8.0 letters and 8.5 letters, resp.), and that bevacizumab administered as needed is equivalent to ranibizumab administered as needed (mean visual acuity increase of 5.9 letters and 6.8 letters, resp.).

## 4. Diagnosis and Initiation of Therapy

### 4.1. Visual Acuity and Clinical Examination

Assessment of patients with suspected neovascular AMD begins with a careful history (especially the nature and duration of symptoms), slit-lamp biomicroscopy, and measurement of visual acuity. Accurate assessment of visual acuity is essential—in this context, log MAR (logarithm of the minimum angle of resolution) visual acuities obtained using Early Treatment Diabetic Retinopathy Study (ETDRS) charts are preferable to Snellen visual acuities (in patients with AMD, Snellen visual acuities are consistently lower (worse) than ETDRS visual acuities) [29]. This factor may be of critical importance in healthcare systems where anti-VEGF therapy can only be provided according to certain prespecified criteria. For example, in the United Kingdom, the NICE (National Institute for Healthcare and Clinical Excellence) guidelines state that patients with log MAR visual acuity ranging between 0.3 (Snellen, 6/12) and 1.2 (Snellen, 6/96) are eligible for treatment [30].

### 4.2. Fluorescein Angiography

Fluorescein angiography is an important component in the initial diagnosis of neovascular AMD and should be carried out in all patients except where precluded by allergy or other systemic considerations [31]. Fluorescein angiography is of particular value for the assessment of CNV lesion location, classification, and size. Determination of CNV location is critical: for well-demarcated lesions located extrafoveally, the use of laser photocoagulation may allow the patient to avoid the need for monthly intravitreous injections over an extended follow-up period [32]. Consideration of angiographic lesion classification is also important when determining whether to initiate treatment. In lesions classified as “occult” on fluorescein angiography, the decision to treat can often be deferred if there is no evidence of disease progression, with many such lesions remaining quiescent for extended time periods [5]. Finally, evaluation of lesion size prior to treatment may provide a useful baseline parameter against which future lesion growth can be assessed, particularly in difficult cases where repeat fluorescein angiography is required [25]. 

Fluorescein angiography may also be useful for the exclusion of other macular disease that can mimic the features of neovascular AMD, such as retinal macroaneurysms resulting in submacular haemorrhage, central serous chorioretinopathy resulting in subretinal and sub-RPE fluid, and pattern dystrophies where there is progressive staining of vitelliform-like material [25]. Fluorescein angiography can also assist in the diagnosis of conditions where CNV is present but due to etiologies other than AMD, for example, angioid streaks, pathologic myopia, inflammatory disorders, trauma [25]. Non-AMD CNV may respond differently to anti-VEGF blockade (e.g., requiring fewer treatments) and, in some case, may benefit from alternative or supplementary treatment regimens (e.g., systemic immunosuppression for inflammatory CNV).

### 4.3. Indocyanine Green Angiography

In many Asian countries, where the prevalence of polypoidal choroidal vasculopathy is high, indocyanine green angiography (ICGA) is an important part of baseline investigations in suspected cases of neovascular AMD [33, 34]. While this is not yet the standard-of-care in most Western countries, baseline ICGA may still be useful in selected cases, particularly in the context of substantial submacular haemorrhage, where it may be useful for determining the presence or extent of any underlying CNV lesion [35].

### 4.4. Signs of Disease Activity

If clinical examination and imaging studies confirm the presence of subfoveal CNV secondary to AMD, and the disease appears active, treatment with intravitreous ranibizumab or bevacizumab should be initiated without delay (treatment delay has been shown to have detrimental effects on final visual outcomes) [36, 37]. In neovascular AMD, these signs of disease activity include

1. deterioration in visual acuity
2. evidence of CNV leakage on fluorescein angiography
3. abnormal retinal thickness on OCT, with evidence of intraretinal, subretinal, or sub-RPE fluid
4. presence/recurrence of intraretinal or subretinal haemorrhage.

In patients presenting with bilateral active CNV lesions, it may be reasonable to treat both eyes in a single setting or within a short time period of each other, providing asepsis is ensured [30, 38].

### 4.5. Other Clinical Scenarios

Patients with neovascular AMD often present with disease features that were not evaluated in—or were specifically excluded from—randomized clinical trials. For example, ranibizumab or bevacizumab therapy may be of benefit in patients with predominantly hemorrhagic lesions, [39, 40] although such lesions were specifically excluded from the pivotal clinical trials of ranibizumab and bevacizumab. The prognosis may be guarded in such patients, however, particularly if there is an underlying RPE tear [41] or if the accumulated blood has had a toxic effect on the RPE and photoreceptors [42]. In addition, some patients with predominantly hemorrhagic CNV lesions may benefit from surgical management approaches, for example, pneumatic displacement of the haemorrhage, with or without the use of tissue plasminogen activator [43]. 

Caution is also required in treating CNV lesions where greater than 50% of the lesion is made up of serous pigment epithelium detachment—such lesions may be at greater risk for development of RPE tears [44]. Other lesions falling outside clinical trial treatment criteria include those with advanced subretinal fibrosis or geographic atrophy involving the foveal center; a trial of bevacizumab or ranibizumab may be indicated but, in general, such cases are unlikely to benefit from continued treatment. Similarly, patients with initial visual acuities worse than 20/320 were excluded from most clinical trials and are less likely to benefit from treatment. Patients with initial visual acuities better than 20/40 are also not typically evaluated in clinical trials of neovascular AMD—treatment of such patients should, however, still be considered in the case of active subfoveal or juxtafoveal disease [45].

Patients with neovascular AMD commonly present with significant ocular comorbidity [30, 38]. In patients with elevated intraocular pressure, even in cases where it exceeds 30 mm of Hg, initiation of antiangiogenic therapy should not be delayed, providing antiglaucoma therapies can be initiated concomitantly. In patients with visually significant cataracts, it is also preferable that CNV activity is controlled prior to any surgical procedure for the removal of cataract.

As the patient population affected by neovascular AMD is, by definition, elderly, the presence of any significant systemic comorbidity is also an important consideration [46]. Bevacizumab was originally developed and approved for the treatment of metastatic colorectal cancer and, in this context, was associated with increased incidences of hypertension, bleeding, and thromboembolic events [21]. While the MARINA and ANCHOR trials did not show any difference in the incidence of serious adverse effects between treatment and control groups, [4, 5]. an interim analysis from the SAILOR study showed a trend for an increase in the incidence of stroke in the group treated with 0.5 mg of ranibizumab [47, 48]. Moreover, the incidence of stroke was higher with preexisting risk factors, in particular a previous history of stroke or arrhythmia. These findings, and recent studies demonstrating both the effects of intravitreous bevacizumab on systemic VEGF levels [49] and an association between AMD itself and higher risk of stroke [50], suggest that extra discussion may be warranted prior to initiation of anti-VEGF treatment in patients with a history of cardiovascular events.

## 5. Retreatment Algorithms and Maintenance Therapy

### 5.1. Fixed Retreatment—Monthly versus Quarterly

In the MARINA and ANCHOR studies of ranibizumab, monthly treatments were provided over a two-year period—each patient thus received a total of 24 injections [4, 5]. Other large clinical trials have since evaluated the benefits of ranibizumab in less-frequent retreatment regimens, for example, the PIER, EXCITE, and SAILOR studies [51–53]. In these studies, patients were initially treated with three injections of ranibizumab on a monthly basis—often termed the loading phase—with subsequent retreatment on a quarterly basis. 

The results of the PIER study, while being generally positive, failed to equal those of the MARINA and ANCHOR trials [51, 52]. Specifically, patients receiving quarterly retreatments had less mean gain in visual acuity, and a smaller percentage of patients experienced 15 or more letters of visual gain. Furthermore, the initial visual gain attained during the loading phase was not maintained during the remainder of the study. 

In the EXCITE study, monthly maintenance therapy with 0.3 mg of intravitreous ranibizumab was compared to the PIER study quarterly regimen (0.3 mg and 0.5 mg) reduced-frequency fixed-dose schedule, with the results again suggesting better mean visual acuity gains in patients receiving monthly retreatment [53]. Finally, the SAILOR study evaluated a loading phase of three consecutive monthly injections followed by quarterly monitoring with retreatment determined by physician discretion (Cohort 2) or by visual acuity and OCT criteria (Cohort 1)—the results of this study provided further confirmation that quarterly visits are insufficient for optimal monitoring of disease progression [54].

### 5.2. “As-Required” Retreatment

Despite the strong evidence from the MARINA and ANCHOR studies, fixed, monthly ranibizumab retreatment regimens have not been widely adopted [5]. While a variety of retreatment protocols are in use, the dosing schedule described in the PrONTO study is perhaps the most widely used in clinical setting [12]. 

In the PrONTO study, an OCT-guided variable dosing regimen was used for the treatment of neovascular AMD with ranibizumab [12]. Subjects enrolled in this small, single-center study received three consecutive monthly intravitreous injections of ranibizumab. After this loading phase, ranibizumab retreatment was performed, in large part, based on OCT criteria: a loss of five letters of visual acuity in conjunction with intraretinal fluid on OCT or an increase of OCT central retinal thickness of at least 100 *μ*m, being indications for retreatment. Other criteria used to trigger retreatment consisted of new-onset haemorrhage or new “classic” CNV (although, for the latter, fluorescein angiography was only required at followup in cases of significant or unexplained visual loss). In the second year of the study, the retreatment criteria were modified so that any evidence of recurrent intraretinal, subretinal, or sub-RPE fluid was grounds for retreatment [55]. The results of this study provided evidence that “as-required” retreatment regimens could be a viable approach when administering ranibizumab in the treatment of neovascular AMD. In the first 12 months of the PrONTO study, participants received an average of 5.6 intravitreous injections of ranibizumab. In contrast, the results from a recent retrospective case series [56], and from a drug and disease model [57], suggest that approximately eight injections may be required, on average, in the first year of individualized retreatment.

The ABC trial, which provided the first level I evidence for the efficacy of bevacizumab in the treatment of neovascular AMD, was also based on an as-required retreatment strategy [27]. In the ABC trial, after an initial loading phase, the criteria for retreatment were persistence or recurrence of subretinal fluid on OCT, new haemorrhage, new classic CNV, or a drop of vision by five or more letters with new intraretinal fluid on OCT. If there was persistent intraretinal fluid after two consecutive treatments, then treatment was withheld assuming that other criteria for retreatment were not triggered. Of note, patients received retreatment, when necessary, at six weekly intervals (in the PrONTO study, monthly followup, and retreatment intervals were used). Using this retreatment approach, the results of the ABC trial demonstrated that, after 54 weeks, 32% of those receiving bevacizumab achieved 15 or more letters of visual gain—results comparable to those reported for fixed retreatment in the MARINA and ANCHOR studies.

More recently, in the large, multicenter, CATT study, the efficacies of ranibizumab and bevacizumab were compared using both fixed, monthly retreatment, and as-required retreatment strategies [28]. In the as-required retreatment groups, the triggers for retreatment included fluid on OCT new or persistent haemorrhage, decreased visual acuity, or dye leakage or increased lesion size on fluorescein angiography (again, fluorescein angiography was only performed at the discretion of the treating physician to aid in retreatment decisions). In the CATT study, ranibizumab administered monthly led to a gain of 8.5 letters at 12 months, whereas ranibizumab administered as needed led to a gain of 6.8 letters. Similarly, bevacizumab administered monthly led to a gain of 8.0 letters at 12 months, whereas bevacizumab administered as needed led to a gain of 5.9 letters. Although fixed monthly retreatment led to greater mean visual acuity gain, the differences were not significant for the ranibizumab groups and were inconclusive for the bevacizumab groups. 

While the results from the ABC and CATT reports are encouraging, both studies have only reported their findings after approximately one year of followup. For longer follow-ups, the results of the SUSTAIN and HORIZON trials have suggested that substantial visual acuity gain is less likely with retreatments that occur less than monthly, whether they be determined on a reduced frequency, fixed dosing schedule, or influenced by imaging parameters such as OCT or fluorescein angiography [25, 28]. For example, the HORIZON extension study demonstrated that one year after entry into the study, when monthly treatments were replaced by physician-determined retreatment decisions median visual acuity decreased by five letters after one year and by a further three letters after two years [25].

### 5.3. “Treat and Extend”

Although there is an absence of evidence from clinical trials, a wide variety of other retreatment regimens—often tailored to healthcare system-specific logistical factors—have been adopted worldwide [58]. In an attempt to minimize both the number of injections required, and the number of hospital visits, a “treat and extend” approach has recently been described [59–61]. In this method, monthly injections with ranibizumab are provided until all signs of exudation have resolved as seen with OCT. The treatment interval is then sequentially lengthened by one to two weeks as long as there are no signs of recurrent exudation. When recurrent exudation is detected on a follow-up visit, the treatment interval is reduced to the prior interval. Thus, treatment is rendered at every visit but the time between visits is individualized based on a given patient's response to treatment. To date, this approach has only been evaluated in the context of single-center retrospective studies.

### 5.4. Neovascular AMD “Refractory” to Treatment

The findings of the PrONTO study suggest that 70% of patients treated with intravitreous ranibizumab will have resolution of macular edema within one month of their first injection (as demonstrated using OCT); similarly, more than 90% of patients will have resolution of all fluid following a loading phase of three consecutive monthly injections [12]. In a small minority of patients, however, the anatomical signs of fluid exudation (and hence disease activity) appear refractory to anti-VEGF therapy—such patients may have polypoidal choroidal vasculopathy, a disease variant best detected using indocyanine green angiography, and common in Asians and African Americans [62, 63]. The treatment of polypoidal choroidal vasculopathy has not yet been adequately addressed in clinical trials, although many authorities recommend the use of anti-VEGF therapy in combination with photodynamic therapy with verteporfin. The results of the EVEREST study—a phase IV clinical trial investigating the use of ranibizumab in PCV—should provide further clarification regarding this [25].

### 5.5. Precision and Safety of Repeated Intravitreous Injection

Repeated intravitreous injection of ranibizumab and bevacizumab, over extended time periods, has been demonstrated to result in a low incidence of serious ocular adverse events. In the CATT study, endophthalmitis developed after only two of 5449 injections (0.04%) in 599 patients treated with ranibizumab, and after only four of 5508 injections (0.07%) in 586 patients treated with bevacizumab. Uveitis, retinal detachment, retinal vascular occlusion or embolism, retinal tear, and vitreous hemorrhage each also occurred in less than 1% of patients. Efforts are underway to reduce further the incidence of these events, with studies evaluating the effect of needle type and injection technique on patient pain levels, vitreal reflux, and ocular complications [64]. Other studies have demonstrated the presence of particulate contaminates—with the risk of inflammatory reaction—following the preparation of bevacizumab for intraocular use [14, 65]; such findings emphasize the need for implementation of stringent protocols when preparing these drugs. Finally, a number of studies have demonstrated significant variations in the accuracy, precision, and repeatability of intravitreal dosages, underlining the need for care in drawing up and administering therapeutics Intravitreously [66].

## 6. Discontinuation of Therapy

Although there is a considerable body of work documenting the natural history of untreated neovascular AMD, the duration of disease activity in patients receiving anti-VEGF therapy is less clear [48]. Similarly, the frequency and likelihood of disease recurrence remains unknown. Evidence from the HORIZON extension study suggests that neovascular AMD may remain active for a number of years—in this study, 61% of patients treated with monthly ranibizumab over a two-year period required further treatment in the third year [25, 48]. Thus, careful monitoring of patients with neovascular AMD, for extended time periods, is mandatory. 

On occasion, discontinuation of treatment for situations other than lack of disease activity may also be required [38, 48]. For example, treatment may be deferred or terminated in situations where further visual improvement appears unlikely, for example, severe RPE tears involving the foveal center, or in the context of significant coexisting geographic atrophy or subfoveal scar formation. Limited evidence exists evaluating the benefits of continued treatment with ranibizumab in the case of RPE tears (although no data suggest that such an approach should be contraindicated).

## 7. Emerging Treatment Strategies and Future Directions

### 7.1. Anti-VEGF Tachyphylaxis

Recent evidence suggests that, while treatment with ranibizumab leads to rapid early reductions in retinal thickness, this effect may be attenuated over time. This attenuation is suggestive of tachyphylaxis (other studies have reported similar findings with regard to bevacizumab) [67–72]. A number of strategies may prove useful in dealing with such phenomenon. Anecdotal evidence suggests that patients exhibiting signs of ranibizumab tachyphylaxis may benefit from being switched to bevacizumab (and vice versa). Similarly, patients with anatomical evidence of tachyphylaxis may benefit from the use of “treatment holidays” or the use of ranibizumab at higher doses. In the ongoing HARBOR trial, OCT-guided therapy is being used to compare the typical dose of ranibizumab (0.5 mg) with an increased dose (2.0 mg). Increased doses of bevacizumab are also under investigation [25, 48].

### 7.2. Role of Combination Therapy

A number of authors have recently suggested that, in neovascular AMD, combining treatments with different mechanisms of action may result in synergistic benefits, including better visual outcomes, reduced frequency of treatments, lower risk of adverse events, and decreased likelihood of “escape” (i.e., the development of alternative pathways by which cells allow themselves to overcome iatrogenic inhibition) [73, 74]. 

To date, much of the work on combination therapies has focused on the use of verteporfin PDT in combination with VEGF blockade [73]. Preliminary results suggest that this approach may reduce the number of treatments required to render the CNV lesion quiescent—but at the expense of achieving maximum visual gain. The 12-month results of the DENALI study were recently reported. In this phase IIIb study, patients receiving combination therapy required fewer retreatments than those receiving ranibizumab monotherapy (2.2 for the standard fluence group and 2.8 for the reduced fluence group, compared with 7.6 additional retreatments in the ranibizumab monotherapy group), but their mean visual acuity gain was less (5.3 letters for the standard fluence group and 4.4 letters for the reduced fluence group, compared with 8.1 letters for the patients receiving ranibizumab monotherapy) [75]. 

VEGF blockade is also being combined with macular radiation therapy, either from an internal approach via introduction of a radioactive probe via pars plana vitrectomy, or from an external approach delivered through the inferior sclera [76]. Preliminary results suggest that epiretinal intraocular radiation, in combination with ranibizumab, may result in a reduced need for intravitreous injections [77]. Caution is still required, particularly with regard to potential ocular adverse effects, and phase III clinical trials are currently underway.

### 7.3. Other Anti-VEGF Therapies—VEGF Trap

Aflibercept (VEGF Trap, Regeneron Pharmaceuticals, Tarrytown, NY) is a fusion protein specifically designed to bind to all isoforms of VEGF with a higher affinity than either bevacizumab or ranibizumab, thereby offering a theoretically longer interval between doses [78, 79]. The use of intravitreous “VEGF Trap-Eye” (a formulation of VEGF Trap for intraocular delivery) has recently been assessed in two phase III clinical trials. In the North American “View 1” trial, patients receiving 2.0 mg of VEGF Trap-Eye—every two months—gained an average of 7.9 letters of visual acuity. Similarly, in the European “View 2” trial, patients receiving 2.0 mg of VEGF Trap-Eye—every two months—gained an average of 8.9 letters of visual acuity (http://www.regeneron.com/vegftrap_eye.html).

### 7.4. Other Therapies

Since the revolutionary introduction of bevacizumab and ranibizumab, much of the clinical focus in neovascular AMD has been on the development of optimal treatment regimens and use of combination therapies. However, it is becoming increasingly clear that the development and progression of CNV lesions occurs in the context of a more slowly evolving, underlying disorder—atrophic or “dry” AMD. It appears likely that, even with early diagnosis and optimal treatment, the visual benefits achievable with anti-VEGF therapies will reach a plateau—and, in some patients, inexorable visual loss may occur. Thus, further visual gains among patients undergoing anti-VEGF therapy may require novel treatment strategies that promote RPE and photoreceptor survival rather than targeting CNV. In this regard, a number of therapies now in development for dry AMD, using neuroprotective, anti-inflammatory, and visual-cycle targeted, strategies, may be of benefit [75].

## 8. Conclusion

The introduction of anti-VEGF therapy was a massive first step in the successful treatment of neovascular AMD and is likely to revolutionize the treatment of many other macular disorders. However, while anti-VEGF therapy represents the current “state of the art” in AMD therapeutics, this situation is likely to change rapidly in the coming years.

## Abbreviations

AMD: Age-related macular degeneration
OCT: Optical coherence tomography
RPE: Retinal pigment epithelium
VEGF: Vascular endothelial growth factor
CNV: Choroidal neovascularization
PDT: Photodynamic therapy.

## Clinical Trial Acronyms

MARINA: Minimally classic/occult trial of the anti-VEGF antibody ranibizumab in the treatment of neovascular AMD
ANCHOR: Anti-VEGF antibody for the treatment of predominantly classic choroidal neovascularization in AMD
CATT: Comparison of age-related macular degeneration treatments trials
ABC: Avastin (bevacizumab) for treatment of choroidal neovascularization
EXCITE: Efficacy and safety of ranibizumab in patients with subfoveal choroidal neovascularization secondary to age-related macular degeneration
SUSTAIN: Study of ranibizumab in patients with subfoveal choroidal neovascularization secondary to age-related macular degeneration
SAILOR: Safety assessment of intravitreal lucentis for AMD
VIEW-VEGF Trap-Eye: Investigation of efficacy and safety in wet age-related macular degeneration.
